# O021: Infection control enclosure (ICE) POD: meeting the need for more single rooms

**DOI:** 10.1186/2047-2994-2-S1-O21

**Published:** 2013-06-20

**Authors:** J Salkeld

**Affiliations:** 1 Bioquell, Andover, UK

## Introduction

An increase in single occupancy rooms reduces transmission, improves hand hygiene compliance and increases patient satisfaction. Thus, there is a demand for more single rooms, particularly in hospitals with a low proportion of single rooms as is common in Europe. Furthermore, where the previous room occupant was infected or colonised with a pathogen, the subsequent admission is significantly less likely to acquire that pathogen if the room is decontaminated using hydrogen peroxide vapour (HPV) rather than being cleaned and disinfected using standard methods.

## Objectives

Bioquell has developed the ‘Infection Control Enclosure’ (ICE)-pod, a bespoke, semi-permanent structure with a door and an integral air handling system to provide a negative air-flow, which can be erected around existing bed spaces without closing wards.

## Methods

The ICE-pod combines many of the benefits of single rooms whilst retaining the primary advantages of bays /open wards. The pods can also be sealed for HPV decontamination.

## Results

ICE-pods can be used to interrupt the spread of nosocomial pathogens by providing additional capacity to segregate patients known to be infected or colonised with pathogens, for pre-emptive segregation of high-risk patients, to provide a single room environment for patients that require high visibility such as those at risk of falls, and the ability to decontaminate individual bed spaces using HPV. The ICE-pod will also improve the privacy and dignity of patients cared for in multi-occupancy areas, and has the potential to “free up” side rooms for patients requiring additional privacy and dignity. There are tangible potential cost-benefit advantages associated with accelerated discharge from intensive care units and other high-cost units and avoiding the high cost of permanent conversion programs. The ICE-pod will provide an overall improvement in the flexibility of patient flow throughout a hospital, which will increase throughput and decrease the number of patients placed temporarily in sub-optimal specialties.

## Conclusion

Implementation is currently underway in the UK and will include assessment of patient and staff acceptability of the ICE-pod. Further trials to evaluate the clinical impact of the ICE-pod are planned.

## Disclosure of interest

J Salkeld: Employee of Bioquell.

